# The use of a modified Delphi approach to engage stakeholders in zoonotic disease research priority setting

**DOI:** 10.1186/1471-2458-14-182

**Published:** 2014-02-20

**Authors:** Kate Sawford, Navneet K Dhand, Jenny-Ann LML Toribio, Melanie R Taylor

**Affiliations:** 1 Farm Animal & Veterinary Public Health, Faculty of Veterinary Science, University of Sydney, Camden, New South Wales, Australia; 2 Centre for Health Research, University of Western Sydney, Penrith, NSW, Australia

**Keywords:** Delphi method, Stakeholder engagement, Zoonotic disease, Hendra virus, Public health, Thematic analysis

## Abstract

**Background:**

After the 2011 cluster of Hendra virus cases in horses in Australia, public health targeted education initiatives at people in the equine industry to reduce human exposure to potentially infected horses. ‘Horse owners and Hendra Virus: A Longitudinal cohort study To Evaluate Risk’ aims to enhance public health measures through improved understanding of Hendra virus risk perception and risk mitigation strategies among horse owners and horse care providers. This paper describes the stakeholder consultation that was undertaken to ensure the cohort study outcomes were relevant to diverse groups who play a role in Hendra virus policy development and implementation.

**Methods:**

A two-round modified Delphi study with online questionnaires was conducted. In round one, stakeholders identified priority research areas. In round two, stakeholders rated and ranked topics that emerged from thematic analysis of the round one responses. Round two data were analysed using logistic regression.

**Results:**

Of the 255 stakeholders contacted, 101 responded to round one. Over 450 topics were proposed. These were organized into 18 themes. Approximately two thirds of the round one respondents participated in round two. ‘Hendra virus-related risk awareness and perception’, ‘personal health and safety’, ‘emergency preparedness’, ‘risk prevention, mitigation, and biosecurity’, and ‘Hendra virus vaccination in horses – attitudes/uptake’ were the top five areas identified according to probability of being ranked extremely important.

**Conclusions:**

In this study, a modified Delphi approach was effective in guiding research into Hendra virus, a zoonotic disease of animal and human health significance. The findings support the notion that stakeholders should be engaged in zoonotic disease research priority setting. Such consultation will help to ensure that research initiatives are relevant and useful to stakeholders in the position to make use of new findings.

## Background

Hendra virus was first isolated from horses in 1994 after an outbreak of severe illness in a racing stable [1]. Two cases of Hendra virus human infection occurred as a result of contact with infected horses, one of which was fatal [1]. From 1994 until 2010 spillover events from flying foxes to horses occurred infrequently and intermittently, with a total of 14 known spillover events in the 16-year period [2]. Though cases were rare, the human and equine case fatality rates were 57% and 75% respectively [2]. However, in 2011 the situation changed dramatically when Hendra virus spilled over from flying foxes into horses 18 times within a 12-week period [3]. While the causes of this cluster of cases in horses remain poorly understood, the public health response has focused on reducing the exposure of people in the equine industry to sick and potentially infected horses and thereby the risk of human infection [4]. Therefore there is a need to engage with ‘horse people’, including individuals who own horses as well as those in regular contact with horses, to understand their perceived vulnerability to Hendra virus, their uptake of recommended risk mitigation strategies, and their unaddressed concerns and fears. This need led to funding of a three-year research project investigating these human dimensions of the Hendra virus challenge titled ‘Horse owners and Hendra Virus: A Longitudinal cohort study To Evaluate Risk’ (The HHALTER project).

The primary research activity proposed within the HHALTER project consists of a series of five online surveys with horse owners and horse care providers. The outcomes from the project will inform strategies to reduce the risk of transmission of Hendra virus from flying foxes to horses, and from horses to humans. There are a number of stakeholder groups that have a role in Hendra virus-related policy development and implementation in Australia, including disease control policy developers, horse industry representatives, researchers, horse health care providers, and wildlife health managers. Therefore, it was necessary to engage with stakeholder groups at the outset of the HHALTER project to ensure the topics addressed by the project surveys would meet the range of needs and priorities of these various groups.

We conducted a modified Delphi study to refine the research priorities of the HHALTER project prior to the primary research activity within the project. The Delphi method allows participants to express their knowledge and viewpoints and then provide feedback on the knowledge and viewpoints put forward by themselves and other participants in a structured and non-confrontational way. Although there are many iterations of the Delphi method [5], typically a series of questionnaires is used with each questionnaire constituting a ‘round’ of data collection. In the first questionnaire participants are asked to identify issues of importance to them. The responses are then analyzed and fed back in a second questionnaire to those who responded in the first round. In the second questionnaire respondents may be asked to revise their original responses or answer other questions based on the responses to the first round from all participants. This process may be repeated any number of times, particularly if the researchers are seeking some level of consensus.

In this instance, a Delphi study was conducted as part of a consultation process with stakeholder groups that influence Hendra virus-related policy development and implementation in Australia to refine the research priorities of the HHALTER project. We demonstrate how this process enabled us to develop and prioritize a list of topics to be addressed by the HHALTER project research activities. Finally, we present information on the similarities and differences between stakeholder groups in terms of the importance they placed on the topics to be addressed by the HHALTER project.

## Methods

### Ethics statement

The ethics committee at the University of Western Sydney approved the study proposal (Protocol No. H9824).

### Study design

A two-round Delphi study was conducted to inform the topics to be addressed by the HHALTER project. It consisted of two online surveys hosted by SurveyMonkey™ with each survey constituting a ‘round’ of data collection. This study was limited to two rounds because we wished to understand priority areas for the project as raised by participants and the differences between the stakeholder groups in terms of their research priorities, but were not seeking consensus on the research topics to be included in the project.

### Sampling

Stakeholders were identified though a number of sources, including the professional contact network of the HHALTER project steering committee, relevant conference proceedings, and stakeholder websites. Individuals contacted included: policy developers and implementers in key government agencies in all states and territories; known experts engaged in a range of Hendra virus-related activities; research leaders in charge of National Hendra Virus Research Program funded projects; members of the Intergovernmental Hendra Virus Taskforce; and public health leaders in Hendra virus-affected states. Those initially contacted via email were asked to identify stakeholders who may have been missed, enabling the research team to identify additional stakeholders.

### Round one questionnaire

A HHALTER project overview and link to the round one questionnaire was distributed to all of the stakeholders using an email collector created in SurveyMonkey™. On page two of the questionnaire participants were advised that “completion of the following questionnaire indicates that you have understood to your satisfaction the information regarding participation in the research project and agree to participate”. Stakeholders were asked to specify the jurisdictional level at which their organization operated. Response options included local, state or federal/national. Those participants whose organization operated at the state level were then asked to specify the state where their organization operated. Finally participants were given the following instruction: ‘please list topic areas relating to horse owners and Hendra virus that you think should be priority areas for questions posed to horse owners in the surveys conducted by the HHALTER project’. Ten separate spaces for listing topic areas were provided. An additional documentation file contains this questionnaire in its entirety (see Additional file 1).

### Development of the round two questionnaire

Responses from round one were analysed and used to construct a round two questionnaire. Working independently, two members of the research team (KS and MRT) read and re-read through the responses to the final question in the questionnaire from round one and coded them using codes that emerged from the data [6]. The aim was to identify concepts, the basic units of analysis in thematic analysis. During the identification of concepts, the central meaning of each suggested topic area was described in a short statement, referred to here as a code. Concepts were grouped into categories, groups of suggested topics that shared common features. Similarly, categories were organized around themes [7]. Thematic analysis allowed KS and MRT to establish categories and themes that occurred throughout the data and organize the data around those themes. They then compared their findings and discussed them at length in order to arrive at an agreed upon list of 18 themes. A desired number of themes was not established at the beginning of data analysis, although it was agreed that if possible the list should not exceed 20 as a number of themes greater than 20 would translate into a questionnaire in round two that would take longer than 15 minutes to complete. This analysis was carried out using Microsoft Excel (Microsoft Office Excel 2011).

### Implementation of the round two questionnaire

The proposed themes and associated categories generated through analysis of the round one data were then presented to those who completed the round one questionnaire in a second questionnaire. In the round two questionnaire themes and categories were referred to as topics and subtopics. Participants were shown each topic and its associated subtopics on a separate page of the questionnaire and asked to rate each topic individually according to rate the importance of each topic area to their role/professional position on a 5-point unipolar scale. Each rating scale was fully labeled with the following labels: ‘not very important’, ‘somewhat important’, ‘moderately important’, ‘important’, and ‘extremely important’. The order in which the topics were presented was randomized for each participant. On the final page of the questionnaire the complete list of topics was shown to participants at which time they were asked to select their top five priority topic areas for the HHALTER project. The ordering of this list was also randomized for each participant. An additional documentation file contains this questionnaire in its entirety (see Additional file 2).

### Statistical data analysis

All statistical analyses were performed using SAS statistical software (release 9.3 © 2002–2008, SAS institute Inc., Cary, NC, USA) and checked using R (version 2.15.2. © 2012, The R Foundation for Statistical Computing) [8]. All figures were produced using Microsoft Excel (Microsoft Office Excel 2011).

### Descriptive analyses

Contingency tables were used to assess the response rate to the round one and round two questionnaires, as well as the overall response rates, for the different stakeholder groups. Contingency tables were also used to examine the relationship between topic ratings and stakeholder group. The distributions of nominal variables, including jurisdictional level and state, were assessed with frequency distributions.

Exploration of the contingency tables for topic ratings revealed that, as expected, responses from participants were skewed toward the upper end of the scale and a large number of cells contained numbers less than five. Therefore the decision was made to create two outcome variables based on the individual topic ratings. To create the first outcome variable, considerably important, the rating categories ‘important’ and ‘extremely important’ were collapsed into a single rating category (considerably important coded 1) and the rating categories ‘not very important’, ‘somewhat important’, and ‘moderately important’ were collapsed into a single rating category (not considerably important coded 0). To create the second outcome variable, extremely important, the rating category ‘extremely important’ was coded 1 and the rating categories ‘not very important’, ‘somewhat important’, ‘moderately important’, and ‘important’ were collapsed into a single rating category (not extremely important coded 0).

### Exploration of the overall rating and ranking of the topic areas

All of the questions that involved rating topics were stacked to create a single variable representing the overall rating for each of the 18 topic areas. This variable was used as an outcome in logistic regression analyses to assess the probability of each question being rated as ‘considerably important’ or ‘extremely important’. An individual stakeholder identification number variable was included as a random effect to account for similarity in responses by a stakeholder. The top five priority areas selected on the final page of the round two questionnaire were coded initially as a binary variable (yes/no) and then all the priority areas were stacked to create a single variable representing priorities for all stakeholders for all questions. Similar analyses as above were conducted to evaluate stakeholders’ ranking of priorities.

### Exploration of the response rates and topic area ratings by stakeholder group

The associations of stakeholder group with the response rate to the round one and round two questionnaires, as well as the overall response rates were assessed in logistic regression analyses.

The association of stakeholder group with each of the binary level of importance outcome variables for each of the proposed topics was assessed in logistic regression analyses. However, even when the rating categories were collapsed to create two binary outcome variables for level of importance there were small values in some of the cells of the contingency tables, including zeros, and as a result the parameter estimates in logistic regression were biased. In order to deal with this separation of data, each of the binary response rate variables was assessed with the logistf package in R that utilizes the penalized maximum likelihood approach developed by Firth [9,10].

## Results

The first questionnaire was sent initially to 216 stakeholders. With additional stakeholders identified by those initially contacted, the final number of stakeholders sent a link to the first questionnaire was 255. Stakeholders came from five stakeholder groups: policy developers and implementers; horse industry representatives; researchers; horse health care providers; and wildlife health managers. Stakeholders from all stakeholder groups provided responses. Around 40 percent (101/255, 39.6%) completed the first questionnaire. Stakeholders who responded to the first questionnaire were sent the second questionnaire. Approximately two thirds of stakeholders who completed the first questionnaire completed the second questionnaire (68/101; 67.3%). Refer to Table 1 for a breakdown of the number of stakeholders contacted and round one, round two, and overall response rates by stakeholder group.

**Table 1:** Sample composition and stakeholder response rates for each round of data collection

	**Number contacted**	**Round 1**		**Round 2**		**Overall proportion**
**Stakeholder group**		**N**	**Proportion**	**n**	**Proportion**	
Policy developers and implementers	62	33	53.2%	27	81.8%	43.6%
Horse industry representatives	124	25	20.2%	11	44.0%	8.9%
Researchers	34	26	76.5%	19	73.1%	55.9%
Horse health care providers	20	9	45.0%	5	55.6%	25.0%
Wildlife health managers	15	8	53.3%	6	75.0%	40.0%
**Total**	255	101	39.6%	68	67.3%	27.4%

Stakeholders from all Australian States and Territories provided responses. Stakeholders operating at the National/Federal level made up over half of respondents (51.5%). Participants operating at the State-level jurisdiction came in large part from Queensland (38.6% of all participants). Table 2 shows the breakdown of participants by jurisdictional level and state.

**Table 2:** Description of the stakeholders who responded to the round one questionnaire

**Variable**	**N**	**Level**	**Frequency**	**Proportion**
Jurisdictional level	101	Federal	52	51.5%
		State	44	43.6%
		Local	5	5.0%
State	44	New South Wales	12	27.3%
		Queensland	17	38.6%
		Victoria	3	6.8%
		South Australia	3	6.8%
		Western Australia	2	4.5%
		Australian Capital Territory	1	2.3%
		Tasmania	2	4.5%
		Northern Territory	4	9.1%

### Thematic analysis of responses to the final question in the round one questionnaire

More than 450 topic areas for the HHALTER project were suggested. These were organized into 18 themes. The themes are listed in Table 3.

**Table 3:** Topics areas and related subtopics that emerged from analysis of the round one questionnaire

**Topic number**	**Topics areas and related subtopics**
1	Risk prevention, mitigation, and biosecurity
	Knowledge of practices to reduce risk of Hendra virus transmission to horses and humans
	Implementation of practices to reduce risk of Hendra virus in horses and humans
	Property and vegetation management as it relates to premise biosecurity
	Enablers and barriers to uptake of recommended behaviours/practices
2	Personal health and safety
	Knowledge of personal risk reduction practices
	Utilization of personal risk reduction practices
	Personal hygiene practices
	Personal protective equipment (PPE) knowledge, availability and use
	Enablers and barriers to utilization of personal health and safety practices
	Measures of risk-related behaviours (close contact with horses, handling of bodily fluids, etc.)
	Concerns about risk to self from other animals (e.g. dogs, wildlife)
3	Hendra virus-related risk awareness and perception
	Vulnerability to Hendra virus (horse(s), self, other people)
	Likelihood of being impacted (horse(s), self, other people)
	Beliefs underlying perceived level of risk
	Fear and concern (horse(s), self, other people)
	Awareness of local risk
	Attitudes towards Hendra virus in the context of other diseases/disease risks
	Perceived risk of Hendra virus relative to other infectious diseases and health threats
4	Hendra virus vaccination in horses – process and implementation
	Process of roll-out, including how best to enable uptake
	Priority horse subpopulations for vaccination
	Persons responsible for administering the vaccine
	Perceived need for compulsory vaccination among horse subpopulations
	Perceived role for government in vaccination
5	Hendra virus vaccination in horses – attitudes and uptake
	Willingness to vaccinate and/or vaccinate regularly
	Anticipated uptake
	Barriers to uptake
	Attitudes toward vaccination including perceived effectiveness and concerns about adverse effects
6	Awareness and knowledge of Hendra virus
	Transmission routes
	Signs and symptoms
	Time between infection and clinical onset of disease
	Time between infection and infectiousness
	Locations and details of previous outbreaks
	Environmental conditions that impact transmission
7	Bats/Flying foxes – attitudes and awareness
	Attitudes to bats/flying foxes
	Attitudes to control of bats/flying foxes
	Awareness of local activity
	Opportunities for interaction with horses
	Protecting horses from bat/flying fox exposure
	Knowledge of the role of bats, bat ecology, and bat feeding and roosting behaviours
8	Communication, information, and education
	Verbal communication with veterinarian(s)/government agencies
	Sources of advice
	Perceived success of government communication
	Role of media
	Desired forms of communication/sources of information
9	Hendra virus surveillance and reporting
	Likelihood of early consideration of Hendra virus
	Response to a sick horse
	Severity of illness in horse(s) before a veterinarian is contacted
	When to notify authorities of a sick horse
	Awareness of reporting responsibilities
	Knowledge of reporting pathways
	Enablers and barriers to reporting of suspect cases
	Concerns about reporting
10	Emergency preparedness
	Expectations and preferences in relation to event management
	Record keeping
	Attitudes toward registration of movements and movement controls
	Recording of horse health status and vaccination history
	Attitudes around horse and horse owner identification
11	Horse health awareness
	Frequency of horse observations
	Monitoring for signs of disease
12	Hendra virus response
	Knowledge of the government response plan
	Expectations of time to diagnosis
	Knowledge of testing and quarantine procedures
	Attitudes to recovered horses
	Knowledge and attitudes toward the issue of Hendra virus recrudescence (i.e. reoccurrence of clinical disease in a previously affected animal or person)
	Knowledge of available support
	Attitudes to government response to cases
	Need for a human vaccine
13	Relationship with veterinarian(s)
	Frequency of consultations and communications
	Health services routinely provided by veterinarians
	Trust in veterinarian(s)
	Health of relationship with veterinarian(s)
14	Responsibility
	Attitudes around who is responsible for Hendra virus risk mitigation and response
	Beliefs concerning who should pay the Hendra virus-related costs
15	Sense of control/effectiveness
	Perceived effectiveness of recommended health and safety and biosecurity practices
	Sense of control over ability to reduce personal risk and risk to other people and animals
16	Information seeking
	Primary source of information
	Preferred sources of information
	Trusted sources of information
	Membership in horse associations
	Access to and use of newsletters/e-alerts/subscriptions
	Use of social media
	Use of social networks/informal word-of-mouth/knowledge sharing
	Attendance at workshops/training
17	Trust
	Trust in government agencies to communicate and respond
	Trust in the research and science informing the Hendra virus response
	Trust in others to report and take the appropriate actions
18	Horse behaviour
	Knowledge and awareness of the behaviour of their horse(s)
	Interactions with wildlife/other domestic species (possums, feral cats, livestock, companion animals)
	Interactions with other horses

### Exploration of the overall rating and ranking of the topic areas

**Figure 1:**
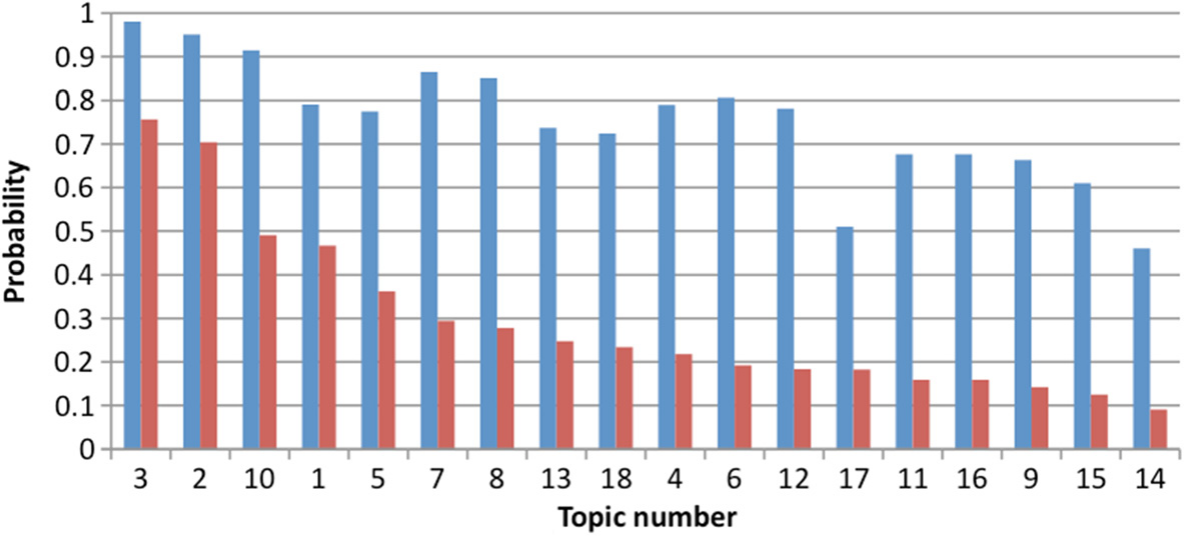
**Probabilities of different topic areas to be ranked as considerably important (blue) or extremely important (red).** The probability of a rating of considerably important (blue) ranged from 0.46 to 0.98 across the topic areas compared to a range of 0.091 to 0.76 for the probability of a rating of extremely important (red).

Figure 1 shows the probability of a topic area being rated as considerably important or extremely important based on logistic regression analyses. There was the greatest range in probabilities across the topics when extremely important remained a separate rating category and therefore the decision was made to sort the topic areas by the probability of a topic area being ranked extremely important.

‘Hendra virus-related risk awareness and perception’, ‘personal health and safety’, ‘emergency preparedness’, ‘risk prevention, mitigation, and biosecurity’, and ‘Hendra virus vaccination in horses – attitudes/uptake’ were the top five topic areas identified according to probability of being ranked extremely important. Logistic regression analyses indicated that the probability of being ranked extremely important by stakeholder groups were significantly different (*p* < 0.001).

**Figure 2:**
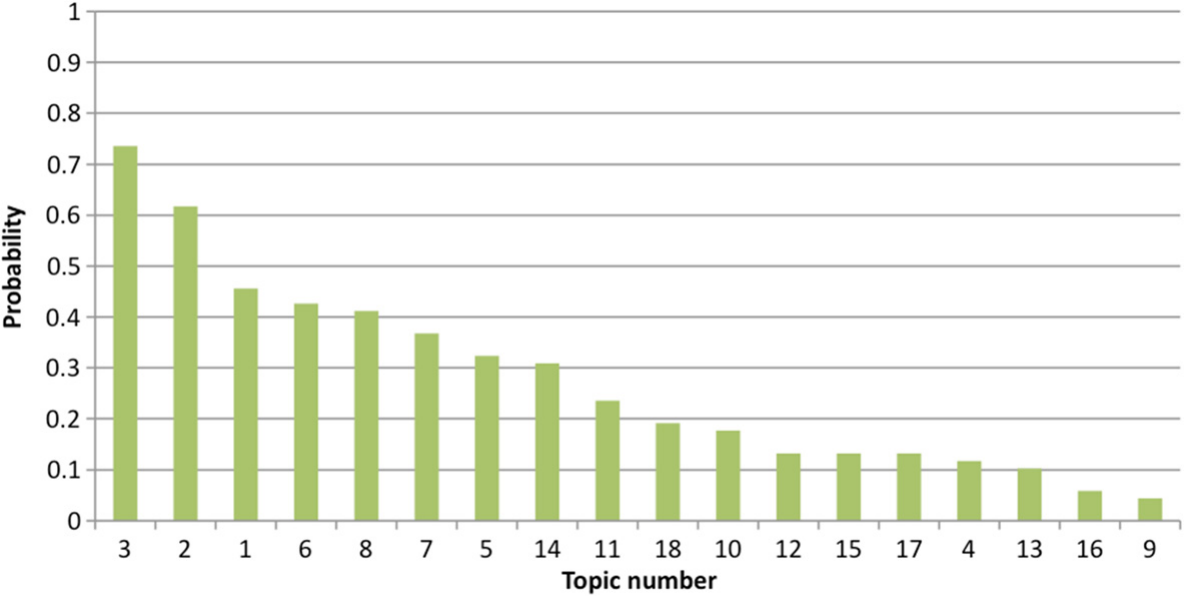
**Probabilities of different topic areas to be nominated as a top five priority area.** The probability of inclusion as a top five priority area ranged from 0.044 to 0.74.

‘Hendra virus-related risk awareness and perception’, ‘personal health and safety’, ‘risk prevention, mitigation, and biosecurity’, ‘awareness and knowledge of Hendra virus’, and ‘communication, information, and education’ were the top five priority areas for research identified by all stakeholders. Logistic regression analyses indicated that the priority areas identified by stakeholder groups were significantly different (*p* < 0.001). Probabilities of different topic areas to be nominated as top five priority areas are presented in Figure 2.

### Exploration of the response rates and topic area ratings by stakeholder group

Horse industry representatives were less likely to respond to the round one questionnaire, the round two questionnaire, and the research project overall. Refer to Table 4 for details of the logistic regression analyses exploring the association between stakeholder group and response rates.

**Table 4:** Contingency tables and logistic regression results for the stakeholder group association with response rates

**Rounds and stakeholder groups**	**Response**						
	**Yes**	**No**	***b***	**SE (*b)***	**Odds ratio**	**95% CI**	***p-value***^***a***^
Round 1							<0.001
Policy developers/implementers	33	29	0.00	-	1.00	-	-
Horse industry representatives	25	99	-1.51	0.34	0.22	0.11, 0.43	-
Researchers	26	8	1.05	0.48	2.86	1.16, 7.65	-
Horse health care providers	9	11	-0.33	0.52	0.72	0.26, 1.98	-
Wildlife health managers	8	7	0.0043	0.58	1.00	0.32, 3.19	-
Round 2							0.034
Policy developers/implementers	27	6	0.00	-	1.00	-	-
Horse industry representatives	11	14	-1.75	0.61	0.18	0.050, 0.55	-
Researchers	19	7	-0.51	0.63	0.60	0.17, 2.09	-
Horse health care providers	5	4	-1.28	0.81	0.28	0.055, 1.40	-
Wildlife health managers	6	2	-0.41	0.93	0.67	0.12, 5.30	-
Overall							<0.001
Policy developers/implementers	27	35	0.00	-	1.00	-	
Horse industry representatives	11	113	-2.07	0.41	0.13	0.055, 0.27	
Researchers	19	15	0.50	0.43	1.64	0.71, 3.86	
Horse health care providers	5	15	-0.84	0.58	0.43	0.13, 1.27	
Wildlife health managers	6	9	-0.15	0.59	0.86	0.26, 2.70	

^a^*p*-values based on likelihood ratio Chi-square test of significance.

Stakeholder groups varied in their rating of the topic area ‘Hendra virus vaccination in horses – attitudes/uptake’ when level of importance was collapsed into the response variables ‘not considerably important’ and ‘considerably important’. Researchers were more likely to rate this topic area as ‘considerably important’ in comparison to the other stakeholder groups. Refer to Table 5 for details of the analysis.

**Table 5:** **Results for the stakeholder group variable associated with the ratings ‘not considerably important’ and ‘considerably important’ (*p*** **< 0.05)**^**a**^

**Topics and stakeholder groups**	**Importance of topic**		***b***	**SE (*b)***	**Odds ratio**	**95% CI**	***p-value***^***b***^
	**Not considerably**	**Considerably**					
Hendra virus vaccination in horses – attitudes/uptake							0.017
Policy developers/implementers	9	18	0	-	1.00	-	-
Horse industry representatives	4	6	-0.30	0.76	0.74	0.18, 3.27	-
Researchers	1	18	1.85	0.96	6.33	1.26, 63.38	-
Horse health care providers	0	5	1.73	1.67	5.65	0.54, 771.14	-
Wildlife health managers	4	2	-1.25	0.94	0.29	0.043, 1.55	-

^a^Topics with *p* > 0.05 not included in this table.

^b^*p*-values based on likelihood ratio Chi-square test of significance.

When level of importance was collapsed into the response variables ‘less important’ and ‘extremely important’, rating of the topic areas ‘Hendra virus-related risk awareness and perception’, ‘Hendra virus vaccination in horses – process and implementation’, Hendra virus vaccination in horses – attitudes/uptake’, ‘Bats/flying foxes – attitudes, awareness’, and ‘Responsibility’ varied between stakeholder groups. Members of all other stakeholder groups were more likely to rate ‘Hendra virus-related risk awareness and perception’ as extremely important compared to policy developers and implementers. Horse health care providers were more likely to rate ‘Hendra virus vaccination in horses – process and implementation’ and ‘Hendra virus vaccination in horses – attitudes/uptake’ as extremely important compared to policy developers and implementers. Wildlife health managers were about 25 times more likely to rate ‘Bats/flying foxes – attitudes, awareness’ as extremely important compared to policy developers and implementers. Finally, researchers were less likely to rate ‘Responsibility’ as extremely important compared to policy developers and implementers. Refer to Table 6 for details of the analysis.

**Table 6:** **Results for the stakeholder group variable associated with the ratings ‘less important’ and ‘extremely important’ (*p*** **< 0.05)**^**a**^

**Topics and Stakeholder groups**	**Importance of topic**		***b***	**SE (*b)***	**Odds ratio**	**95% CI**	***p*-value**^**b**^
	**Less**	**Extremely**					
Hendra virus-related risk awareness and perception							0.026
Policy developers/implementers	21	6	0	-	1.00	-	-
Horse industry representatives	4	6	1.56	0.79	4.78	1.11, 22.83	-
Researchers	8	11	1.50	0.65	4.48	1.33, 16.47	-
Horse health care providers	1	4	2.30	1.13	9.92	1.49, 113.38	-
Wildlife health managers	2	4	1.78	0.97	5.95	1.06, 41.56	-
Hendra virus vaccination in horses – process and implementation							0.026
Policy developers/implementers	25	2	0	-	1.00	-	-
Horse industry representatives	6	5	2.16	0.91	8.63	1.66, 58.16	-
Researchers	15	3	0.83	0.91	2.30	0.40, 15.23	-
Horse health care providers	2	3	2.66	1.13	14.28	1.92, 139.11	-
Wildlife health managers	4	2	1.74	1.09	5.67	0.71, 47.48	-
Hendra virus vaccination in horses – attitudes/uptake							0.025
Policy developers/implementers	21	6	0	-	1.00	-	-
Horse industry representatives	5	5	1.20	0.78	3.31	0.76, 15.10	-
Researchers	12	7	0.69	0.66	1.98	0.56, 7.22	-
Horse health care providers	0	5	3.59	1.68	36.38	3.41, 5024.17	-
Wildlife health managers	4	2	0.61	0.97	1.84	0.27, 10.69	-
Bats/flying foxes – attitudes, awareness							0.0063
Policy developers/implementers	24	3	0	-	1.00	-	-
Horse industry representatives	6	4	1.58	0.87	4.85	0.95, 27.37	-
Researchers	13	6	1.22	0.76	3.37	0.82, 16.21	-
Horse health care providers	2	3	2.28	1.08	9.80	1.42, 82.61	-
Wildlife health managers	1	5	3.25	1.15	25.67	3.71, 317.04	-
Responsibility							0.019
Policy developers/implementers	18	9	0	-	1.00	-	-
Horse industry representatives	6	4	0.30	0.76	1.35	0.31, 5.67	-
Researchers	19	0	-2.00	1.53	0.050	0.00038, 0.44	-
Horse health care providers	4	1	-0.43	1.11	0.65	0.059, 4.22	-
Wildlife health managers	6	0	-1.90	1.64	0.15	0.0011, 1.51	-

^a^Topics with *p* > 0.05 not included in this table.

^b^*p*-values based on likelihood ratio Chi-square test of significance.

## Discussion

In responding to the request for participation, the stakeholders contacted demonstrated a willingness to participate in the process of refining the areas for research within the HHALTER project. Moreover, study participants provided a large number of topic suggestions in the round one survey. This finding supports the notion that stakeholders in public health issues such as zoonotic emerging infectious diseases have ideas and opinions about the need for further research, and given their stake in the issue and expertise, they should be engaged in setting research priorities. The suggestions in round one were organized around 18 broad themes, and then rated and ranked in round two. The findings from this process have already served to inform the research activities within the HHALTER project, and will continue to do so for the remaining duration of the project.

The Delphi method is used frequently in a variety of fields, including health research, as a structured way of soliciting viewpoints from a range of individuals on a specific complex problem or issue. In the health field it has been used to inform research priorities, particularly when the research has implications for a variety of stakeholder groups [11]. There are a number of advantages to the Delphi method: it allows for inclusion of a large number of individuals over a wide geographical area; respondents can take the time they deem necessary to consider the questions before providing responses; and participants provide individual responses anonymously which guards against particular individuals directing the process [12]. Disadvantages can include: low response rates; a lack of dialogue and collaboration between participants that can help foster new ideas; and participant fatigue because of the requirement to complete multiple surveys [12].

This paper describes in detail the use of a modified Delphi approach to guide research priority setting related to a zoonotic emerging infectious disease. Considerable effort was made to invite all stakeholders with a role in Hendra virus policy development and implementation to participate, however it is impossible to know if the sampling strategy was effective in achieving this goal. Efforts to improve response rates across the stakeholders groups would enhance the internal validity of future similar studies, while efforts to sample larger numbers of stakeholders have the potential to improve external validity. Researchers had the highest overall response rate, followed by policy developers and implementers, wildlife health managers, horse health care providers, and horse industry representatives. This ordering of stakeholder groups may be a reflection of each stakeholder group’s comfort level with the research process: one would expect researchers and policy developers and implementers to be more familiar with the research process compared to horse industry representatives. Logistic regression analysis indicated that horse industry representatives were significantly less likely to respond to each of the questionnaires, and the stakeholder consultation process overall. In future, a different approach may be needed to improve engagement with this sector in research priority setting. This group may not be as willing to participate in an online research initiative, and face-to-face or telephone interviews, or a mixed mode design may be a more appropriate alternative [13]. However, there are issues that need to be considered around mode of data collection and measurement issues before adopting this type of approach [13]. Perhaps engaging with this group prior to issuing an invitation would have built trust and encouraged a higher response rate.

For some of the topic areas, ratings differed across the stakeholder groups. Some of these differences are easily understood, while others are not. Hendra virus represents an occupational hazard to horse health care providers, and therefore it is not surprising that members of this group were more likely to rate topics related to Hendra virus vaccination in horses as extremely important compared to policy developers and implementers. Similarly, wildlife health managers may have an advisory role in flying fox protection and management, in addition to direct responsibilities, and therefore it is not surprising that members of this group were more likely to rate ‘Bats/flying foxes – attitudes, awareness’ as extremely important compared to policy developers and implementers. Finally, researchers have less of a stake in issues around Hendra virus responsibility compared to policy developers and implementers, and this may explain why they were less likely to rate ‘Responsibility’ as extremely important compared to policy developers and implementers. In contrast, it is difficult to rationalize why members of all other stakeholder groups were more likely to rate ‘Hendra virus-related risk awareness and perception’ as extremely important compared to policy developers and implementers. Perhaps policy developers and implementers are more familiar with reviewing and interpreting information from the natural sciences, and less so with research from the social sciences, and the considerable significance it has for government policy. This difference warrants further investigation.

The lowest point on the rating scale, ‘not very important’, was set intentionally as a non-zero point. This decision was made because the stakeholders themselves identified the topics and therefore few responses at the lower end of the scale were expected, an effect that would be enhanced were the lowest point on the scale set to zero. In spite of this decision, responses were still skewed toward the upper end of the scale. This characteristic of the data meant that even when the rating categories were collapsed to create dichotomous outcome variables, some of the cells of the contingency tables contained small values, and therefore logistic regression analysis needed to be carried out using the penalized maximum likelihood approach [9,10]. While this approach was necessary due to the characteristics of the data, the penalized maximum likelihood approach is a more conservative test compared to logistic regression where this correction is not applied. While a more conservative approach decreases the probability of incorrectly finding a difference between the stakeholder groups when none exists, it does mean that true differences in the ranking of topics by stakeholder group may have been missed. Future stakeholder consultation processes that employ the method used here need to keep this limitation in mind during study design. Ways to increase cell number size could include altering the rating scale to decrease the amount of skew in the data and ensuring that sufficient numbers of stakeholders from each of the stakeholder groups are contacted and participate in the study.

It is interesting to note that the ordering of topic areas differed depending on whether they were sorted according to probability of being ranked extremely important or probability of being nominated as a top five priority area for research. In guiding the research priorities of the HHALTER project, we have chosen to use the ranking according to probability of being ranked extremely important. We believe this ranking to be more appropriate in guiding cohort survey content because it reflects participants consideration of each topic and its associated subtopics in isolation of other topics and subtopics, though importance rating may have been influenced by the topics and subtopics that came before. Also, we have the ability to include questions on all of the proposed topics in the cohort surveys, but then vary the number of questions according to topic ranking. Research projects that are limited in terms of the number of topics they can address may place more emphasis on probability of being nominated as a top five priority area for research. Future stakeholder consultation processes should ensure that responses to the questions asked will provide information that can best be used to inform the research to follow. For example, rather than select the top five priority areas, other consultation processes may wish to ask the participants themselves to rank a list of proposed topics on a numerical scale.

## Conclusions

Research with the overarching goal to improve public health needs to consider the priorities of stakeholders in the position to act on new findings. In the case of zoonotic emerging infectious diseases with a wildlife reservoir, such as Hendra virus, there are a number of stakeholder groups whose perspective should be incorporated into research priority setting. The modified Delphi approach employed in this study is one way to take into account viewpoints from a range of stakeholders. Undertaking a consultation process that ensures this information gathering happens prior to undertaking any public health research initiative will help to generate research findings that are relevant, usable, and contextually appropriate.

## Competing interests

The authors declare that they have no competing interests.

## Authors’ contributions

KS designed the study, performed the data analysis, and drafted the manuscript. NKD performed the statistical analysis and helped to draft the manuscript. JALMLT participated in study design and coordination. MRT conceived of the study, and participated in its design and coordination. All authors read and approved the final manuscript.

## Pre-publication history

The pre-publication history for this paper can be accessed here:

http://www.biomedcentral.com/1471-2458/14/182/prepub

## Supplementary Material

Additional file 1 Round one questionnaire – the first questionnaire distributed to all stakeholders identified during sampling. Click here for file

Additional file 2 Round two questionnaire – the second questionnaire distributed to those stakeholders who completed the round one questionnaire. Click here for file
