# Supplementary material for: The use of a modified Delphi approach to engage stakeholders in zoonotic disease research priority setting
Source: BMC Public Health. 2014 Feb 20;14:182. doi: 10.1186/1471-2458-14-182 (PMC4015955; doi:10.1186/1471-2458-14-182)
Supplement: Additional file 1 — Round one questionnaire – the first questionnaire distributed to all stakeholders identified during sampling. [file 1471-2458-14-182-S1.pdf]

Introduction

Project overview

HHALTER – Horse owners and Hendra virus: A Longitudinal cohort study To Evaluate Risk

HHALTER is a three-year project funded under the National Hendra Virus Research Program through the Australian, Queensland and New South Wales governments. Researchers at The University of Western Sydney, The University of Sydney, Biosecurity Queensland, and New South Wales Department of Primary Industries are directing the project.

The overarching goal of the project is to inform strategies that reduce the transmission of Hendra virus from flying foxes to horses and from horses to humans.

Central to the HHALTER study will be a large and inclusive cohort of horse owners and horse care providers that will be studied over a two-year period. During the first few months of the project horse owners and horse care providers from all sectors of the horse industry will be invited to join this cohort and take part in the project. This project aims to attract in excess of 2500 people.

The study will investigate factors influencing Hendra virus risk awareness and uptake of risk mitigation practices among horse owners and horse care providers, as well as their attitudes and opinions about a range of related issues. Five cohort surveys will be conducted at six-month intervals over a 24-month period with the first survey due for release toward the end of October 2012.

In order to develop survey content that is relevant to a range of stakeholders we are pursing a consultation process with experts from a variety of disciplines and organizations with a range of roles and responsibilities. The aim is to identify keys issues and current or anticipated concerns/challenges that need to be included in this research. This will inform the list of topics that will be addressed by core questions in the HHALTER survey every six months, and additional topics that might be included as supplementary questions in a select number of surveys over the course of the project.

This stakeholder consultation process will consist of two phases:
Phase 1 - In this first phase, presented here, we are asking you to identify priority research areas for the cohort study.
Phase 2 - We will amalgamate the responses to Phase 1 and then send them out to respondents as part of a follow-up survey. You will then be asked to rate each area in terms of its research priority and have the opportunity to comment.

Your input to each phase should take no longer than 10 minutes to complete. We believe this process of stakeholder consultation is critical for HHALTER to generate information that is applicable and relevant to the challenges posed by Hendra virus, in addition to future infectious disease outbreaks.

We would be extremely grateful for your participation.

Important information

The University of Western Sydney Human Research Ethics Committee has approved the HHALTER project proposal (Protocol No. H9824). If you have any concerns about the ethical conduct of this research you may contact the Ethics Committee through the Office of Research Services on Tel (02) 4736 0083, Fax (02) 4736 0013, or email humanethics@uws.edu.au. Any issues you raise will be treated in confidence and investigated fully, and you will be informed of the outcome.

The identity of respondents will be kept strictly confidential. Only researchers directly involved with the HHALTER project will have access to the responses provided. We intend to publish the results of the HHALTER project in the primary literature. During the process of data analysis questionnaire responses will be aggregated. We may also present individual responses but the identify of the respondent associated with a particular piece of data will remain strictly anonymous (i.e. we will not link personal identifiers to responses during manuscript preparation).

Participation is entirely voluntary: you are not obliged to be involved. Your completion of the following questionnaire indicates that you have understood to your satisfaction the information regarding participation in the research project and agree to participate. Questionaire completion also indicates that you consent to the research group contacting you again with a link to the second questionnaire. In no way does this waive your legal rights nor release the investigators, or involved institutions from their legal and professional responsibilities.

If you have any questions about this study please contact the principal researcher, Dr. Melanie Taylor at melanie.taylor@uws.edu.au or (02) 9685 9552.

Stakeholder group membership

*1. Which stakeholder group do you primarily represent?

- Disease control policy developers and implementers
- Elected officials
- Horse industry representatives
- Human health professionals
- Public health practitioners
- Researchers
- Veterinary practitioners
- Other horse health care providers (equine dentists, massage therapists, farriers, nutritionists)
- Wildlife health managers
- Other (please specify)

*2. At what jurisdictional level does your organization operate?

State or Territory

***3. In which state or territory of Australia does your organization operate?**

- ◯ ACT
- ◯ NSW
- ◯ NT
- ◯ QLD
- ◯ SA
- ◯ TAS
- ◯ VIC
- ◯ WA

Topics to be addressed by the HHALTER project

***4. Please list topic areas relating to horse owners and Hendra virus that you think should be priority areas for questions posed to horse owners in the surveys conducted by the HHALTER project.**

Topic area 1

Topic area 2

Topic area 3

Topic area 4

Topic area 5

Topic area 6

Topic area 7

Topic area 8

Topic area 9

Topic area 10

5. If you require additional space to list more topic areas or make any comments please use the text box below.

Thank you

Thank you for completing phase one of the HHALTER Project Stakeholder Consultation. Your efforts are sincerely appreciated.

We will email you a link to the second survey in 10 to 14 days time.
