# Supplementary material for: The use of a modified Delphi approach to engage stakeholders in zoonotic disease research priority setting
Source: BMC Public Health. 2014 Feb 20;14:182. doi: 10.1186/1471-2458-14-182 (PMC4015955; doi:10.1186/1471-2458-14-182)
Supplement: Additional file 2 — Round two questionnaire – the second questionnaire distributed to those stakeholders who completed the round one questionnaire. [file 1471-2458-14-182-S2.pdf]

Overview

This questionnaire marks the second and final phase of the Stakeholder Consultation process for HHALTER – Horse owners and Hendra virus: A Longitudinal cohort study To Evaluate Risk.

In the first survey we gathered data from stakeholders on priority research areas that could be investigated by the HHALTER project. We received feedback from more than 90 stakeholders who suggested over 450 topic areas related to horse owners and Hendra virus.

We analysed these data to condense the list of potential topics into 18 main themes. With this type of analysis some overlap between themes is inevitable, the proposed topics are meant to encompass all the suggested research topics while keeping redundancy to a minimum.

In this questionnaire each topic area, with a list of sub-topics, will be presented on a separate page. We would like you to rate the importance of each topic area to you in your role/professional position from 'not very important' to 'extremely important'. If you wish you may comment on each topic area.

On the last page of the questionnaire you will be shown a list of all the topic areas and you will be asked to select your top five priority areas for the HHALTER project.

This final stage of the consultation process is extremely important to the HHALTER project, and will help us to prioritise areas in the research. We would be extremely grateful for your participation.

Important information

The University of Western Sydney Human Research Ethics Committee has approved the HHALTER project proposal (Protocol No. H9824). If you have any concerns about the ethical conduct of this research you may contact the Ethics Committee through the Office of Research Services on Tel (02) 4736 0083, Fax (02) 4736 0013, or email humanethics@uws.edu.au. Any issues you raise will be treated in confidence and investigated fully, and you will be informed of the outcome.

The identity of respondents will be kept strictly confidential. Only researchers directly involved with the HHALTER project will have access to the responses provided. We intend to publish the results of the HHALTER project in the primary literature. During the process of data analysis questionnaire responses will be aggregated. We may also present individual responses but the identify of the respondent associated with a particular piece of data will remain strictly anonymous (i.e. we will not link personal identifiers to responses during manuscript preparation).

Participation is entirely voluntary: you are not obliged to be involved. Your completion of the following questionnaire indicates that you have understood to your satisfaction the information regarding participation in the research project and agree to participate. Questionaire completion also indicates that you consent to the research group contacting you again with a link to the second questionnaire. In no way does this waive your legal rights nor release the investigators, or involved institutions from their legal and professional responsibilities.

If you have any questions about this study please contact the principal researcher, Dr. Melanie Taylor at melanie.taylor@uws.edu.au or (02) 9685 9552.

Topic to be addressed by the HHALTER project

Hendra virus-related risk awareness and perception

Vulnerability to Hendra virus (horse(s), self, other people)
Likelihood of being impacted (horse(s), self, other people)
Beliefs underlying perceived level of risk
Fear and concern (horse(s), self, other people)
Awareness of local risk
Attitudes towards Hendra virus in the context of other diseases/disease risks
Perceived risk of Hendra virus relative to other infectious diseases and health threats

Not very important	Somewhat important	Moderately important	Important	Extremely important
○	○	○	○	○

Comments

Topic to be addressed by the HHALTER project

Personal health and safety

Knowledge of personal risk reduction practices
Utilization of personal risk reduction practices
Personal hygiene practices
Personal protective equipment (PPE) knowledge, availability and use
Enablers and barriers to utilization of personal health and safety practices
Measures of risk-related behaviours (close contact with horses, handling of bodily fluids, etc)
Concerns about risk to self from other animals (e.g. dogs, wildlife)

Not very important	Somewhat important	Moderately important	Important	Extremely important
○	○	○	○	○

Comments

Topic to be addressed by the HHALTER project

Risk prevention and mitigation, and biosecurity

Knowledge of practices to reduce risk of Hendra virus transmission to horses and humans
Implementation of practices to reduce risk of Hendra virus in horses and humans
Property and vegetation management as it relates to premise biosecurity
Enablers and barriers to uptake of recommended behaviours/practices

Not very important	Somewhat important	Moderately important	Important	Extremely important
○	○	○	○	○

Comments

Topic to be addressed by the HHALTER project

Sense of control/effectiveness

Perceived effectiveness of recommended health and safety and biosecurity practices Sense of control over ability to reduce personal risk and risk to other people and animals

Not very important	Somewhat important	Moderately important	Important	Extremely important
○	○	○	○	○

Comments

Topic to be addressed by the HHALTER project

Hendra virus vaccination in horses – attitudes/uptake

Willingness to vaccinate and/or vaccinate regularly
Anticipated uptake
Attitudes toward vaccination including perceived effectiveness and concerns about adverse effects
Barriers to uptake

Not very important	Somewhat important	Moderately important	Important	Extremely important
○	○	○	○	○

Comments

Topic to be addressed by the HHALTER project

Hendra virus vaccination in horses – process and implementation

Process of roll-out, including how best to enable uptake

Priority horse subpopulations for vaccination

Persons responsible for administering the vaccine

Perceived need for compulsory vaccination among horse subpopulations

Perceived role for government in vaccination

Not very important	Somewhat important	Moderately important	Important	Extremely important
◯	◯	◯	◯	◯

Comments

Topic to be addressed by the HHALTER project

Bats/Flying foxes – attitudes, awareness, and knowledge

Attitudes to bats/flying foxes

Attitudes to control of bats/flying foxes

Awareness of local activity

Opportunities for interaction with horses

Protecting horses from bat/flying fox exposure

Knowledge of the role of bats, bat ecology, and bat feeding and roosting behaviours

Not very important	Somewhat important	Moderately important	Important	Extremely important
○	○	○	○	○

Comments

Topic to be addressed by the HHALTER project

Awareness and knowledge of Hendra virus

Transmission routes

Signs and symptoms

Time between infection and clinical onset of disease

Time between infection and infectiousness

Locations and details of previous outbreaks

Environmental conditions that impact transmission

Not very important	Somewhat important	Moderately important	Important	Extremely important
○	○	○	○	○

Comments

Topic to be addressed by the HHALTER project

Horse health awareness

Frequency of horse observations

Monitoring for signs of disease

Not very important	Somewhat important	Moderately important	Important	Extremely important
○	○	○	○	○

Comments

Topic to be addressed by the HHALTER project

Hendra virus surveillance and reporting

Likelihood of early consideration of Hendra virus

Response to a sick horse

Severity of illness in horse(s) before a veterinarian is contacted

When to notify authorities of a sick horse

Awareness of reporting responsibilities

Knowledge of reporting pathways

Enablers and barriers to reporting of suspect cases

Concerns about reporting

Not very important	Somewhat important	Moderately important	Important	Extremely important
○	○	○	○	○

Comments

Topic to be addressed by the HHALTER project

Hendra virus response

Knowledge of the government response plan
Expectations of time to diagnosis
Knowledge of testing and quarantine procedures
Attitudes to recovered horses
Knowledge and attitudes toward the issue of Hendra virus recrudescence (i.e. reoccurrence of clinical disease in a previously affected animal or person)
Knowledge of available support
Attitudes to government response to cases
Need for a human vaccine

Not very important	Somewhat important	Moderately important	Important	Extremely important
○	○	○	○	○

Comments

Topic to be addressed by the HHALTER project

Information seeking

Primary source of information

Preferred sources of information

Trusted sources of information

Membership in horse associations

Access to and use of newsletters/e-alerts/subscriptions

Use of social media

Use of social networks/informal word-of-mouth/knowledge sharing

Attendance at workshops/training

Not very important	Somewhat important	Moderately important	Important	Extremely important
○	○	○	○	○

Comments

Topic to be addressed by the HHALTER project

Communication, information, and education

Verbal communication with veterinarian(s)/government agencies
Sources of advice
Perceived success of government communication
Role of media
Desired forms of communication/sources of information

Not very important	Somewhat important	Moderately important	Important	Extremely important
○	○	○	○	○

Comments

Topic to be addressed by the HHALTER project

Horse behaviour

Knowledge and awareness of the behaviour of their horse(s)
Interactions with wildlife/other domestic species (possums, feral cats, livestock, companion animals)
Interactions with other horses

Not very important	Somewhat important	Moderately important	Important	Extremely important
○	○	○	○	○

Comments

Topic to be addressed by the HHALTER project

Relationship with veterinarian(s)

Frequency of consultations and communications

Health services routinely provided by veterinarians

Trust in veterinarian(s)

Health of relationship with veterinarian(s)

Not very important	Somewhat important	Moderately important	Important	Extremely important
○	○	○	○	○

Comments

Topic to be addressed by the HHALTER project

Trust

Trust in government agencies to communicate and respond

Trust in research and science informing the Hendra virus response

Trust in other people to report and take the appropriate actions

Not very important	Somewhat important	Moderately important	Important	Extremely important
○	○	○	○	○

Comments

Topic to be addressed by the HHALTER project

Responsibility

Attitudes around who is responsible for Hendra virus risk mitigation and response

Beliefs concerning who should pay the Hendra virus-related costs

Not very important	Somewhat important	Moderately important	Important	Extremely important
○	○	○	○	○

Comments

Topic to be addressed by the HHALTER project

Emergency preparedness

Expectations and preferences in relation to event management

Record keeping

Attitudes toward registration of movements and movement controls

Recording of horse health status and vaccination history

Attitudes around horse and horse owner identification

Not very important	Somewhat important	Moderately important	Important	Extremely important
○	○	○	○	○

Comments

List of proposed topics

Please select the top FIVE topic areas relating to horse owners and Hendra virus that you think should be priority areas for questions posed to horse owners in the surveys conducted by the HHALTER project.

- [ ] Trust
- [ ] Horse behaviour
- [ ] Relationship with veterinarian/s
- [ ] Horse health awareness
- [ ] Hendra virus-related risk awareness and perception
- [ ] Information seeking
- [ ] Sense of control/ effectiveness
- [ ] Communication, information, and education
- [ ] Vaccination – process and implementation
- [ ] Emergency Preparedness
- [ ] Personal health and safety
- [ ] Responsibility
- [ ] Vaccination – attitudes/uptake
- [ ] Hendra virus response
- [ ] Bats/Flying foxes – attitudes, awareness, and knowledge
- [ ] Awareness and knowledge of Hendra virus
- [ ] Risk prevention and mitigation, and biosecurity
- [ ] Hendra virus surveillance and reporting

Comments

Thank you

Thank you for completing this final phase of the HHALTER Project Stakeholder Consultation. Your input is sincerely appreciated.
